# USP22 knockdown enhanced chemosensitivity of hepatocellular carcinoma cells to 5-Fu by up-regulation of Smad4 and suppression of Akt

**DOI:** 10.18632/oncotarget.15798

**Published:** 2017-03-01

**Authors:** Jing Zhang, Nan Luo, Yu Tian, Jiazhi Li, Xiaozhou Yang, Huimin Yin, Congshu Xiao, Jie Sheng, Yang Li, Bo Tang, Rongkuan Li

**Affiliations:** ^1^ Department of Infection, The Second Hospital of Dalian Medical University, Dalian, Liaoning, P.R. China; ^2^ Division of Hepatobiliary and Pancreatic Surgery, Department of Surgery, The Second Hospital of Dalian Medical University, Dalian, Liaoning, P.R. China; ^3^ Department of Pathology, Dalian Medical University, Dalian, Liaoning, P.R. China; ^4^ Department of Urology, The Second Hospital of Dalian Medical University, Dalian, Liaoning, P.R. China

**Keywords:** USP22, liver neoplasms, chemoresistance, Smad4, Akt

## Abstract

USP22, a member of the deubiquitinases (DUBs) family, is known to be a key subunit of the human Spt-Ada-Gcn5 acetyltransferase (hSAGA) transcriptional cofactor complex. Within hSAGA, USP22 removes ubiquitin from histone proteins, thus regulating the transcription and expression of downstream genes. USP22 plays important roles in many cancers; however, its effect and the mechanism underlying HCC chemoresistance remain unclear. In the present study, we found that USP22 was highly expressed in chemoresistant HCC tissues and cells and was correlated with the prognosis of HCC patients who received chemotherapy. Silencing USP22 in chemoresistant HCC Bel/Fu cells dramatically inhibited proliferation, migration, invasion and epithelial-mesenchymal transition *in vitro*; suppressed tumorigenic and metastatic capacities *in vivo*; and inhibited drug resistance-related proteins (MDR1, LRP, MRP1). Mechanistically, we found that USP22 knockdown exerts its function through down-regulating PI3K and activating Smad4, which inhibited phosphorylation of Akt. Silencing Smad4 blocked USP22 knockdown-induced Akt inhibition in Bel/Fu cells. Our results, for the first time, provide evidence that USP22 plays a critical role in the development of chemoresistant HCC cells and that high USP22 expression serves as a molecular marker for the prognosis of HCC patients who undergo chemotherapy.

## INTRODUCTION

Hepatocellular carcinoma (HCC) is one of the most common malignancies and accounts for the second highest cancer-related mortality. There are approximately 800,000 new cases of liver cancer and 750,000 deaths worldwide per year. There is a high incidence of HCC in China, with China alone accounting for approximately 50% of the total number of cases and deaths [1, 2]. Liver resection, transplantation and percutaneous radiofrequency ablation (PRFA) are applicable treatment options for a portion of HCC patients; the majority of advanced HCC patients still require systemic chemotherapy [3]. However, the development of chemoresistance occurs in most HCC patients, leading chemotherapy failure [4].

Ubiquitin-specific protease 22 (USP22) is a member of the deubiquitinases (DUBs) family. USP22 consists of 525 amino acids, and there is a putative ubiquitin hydrolase containing a C-terminal peptidase domain and an N-terminal UBP-type zinc finger motif that mediates the association of these enzymes with other proteins [5–7]. In addition, as a crucial subtype of human Spt-Ada-Gcn5 acetyltransferase (hSAGA), USP22 promotes the stability of multiple cancer-associated protein targets through deubiquitylation and influences oncogene accumulation [7, 8]. Furthermore, USP22 has been considered to be a proto-oncogene because its expression is significantly upregulated in malignant tumors of several tissues, including the cervix, colon and liver [9, 10], and it participates in regulating proliferation, metastasis and recurrence [6].

Multidrug resistance (MDR) of tumor cells is characterized by resistance to a variety of different structures and different functions or to cytotoxic chemotherapeutic agents. MDR alters the microenvironment and decreases the efficacy of cytotoxic agents, leading to increased DNA repairation, decreased cell apoptosis and changes in drug metabolism [11, 12]. In general, the most important mechanism underlying MDR is the overexpression of the adenosine triphosphate (ATP)-binding cassette (ABC) super-family of transporters, which efflux both cytotoxic agents and targeted anticancer drugs using ATP driven energy [13]. P-gp, LRP and MPR1 are reported to be MDR-related genes. Up-regulation of these proteins promotes chemoresistance, whereas down-regulation of them makes tumor cells more sensitive to chemotherapeutic agents [14, 15]. Moreover, the epithelial-mesenchymal transition (EMT) is also regarded to be an important pathological process in acquired resistance [16]. Therefore, clarification of the causes and mechanism of MDR is very important for HCC patients undergoing chemotherapy.

In this study, we found that USP22 was overexpressed in chemoresistant HCC tissues, indicating that USP22 might be correlated with the development of chemoresistance in HCC. In addition, we present evidence that USP22 promotes Bel/Fu cell growth, migration, invasion, EMT and chemoresistance. These functional effects of USP22 were exerted through up-regulation of Smad4 and suppression of Akt.

## RESULTS

### Aberrant expression of USP22 in HCC was correlated with chemoresistance

**Figure 1:**
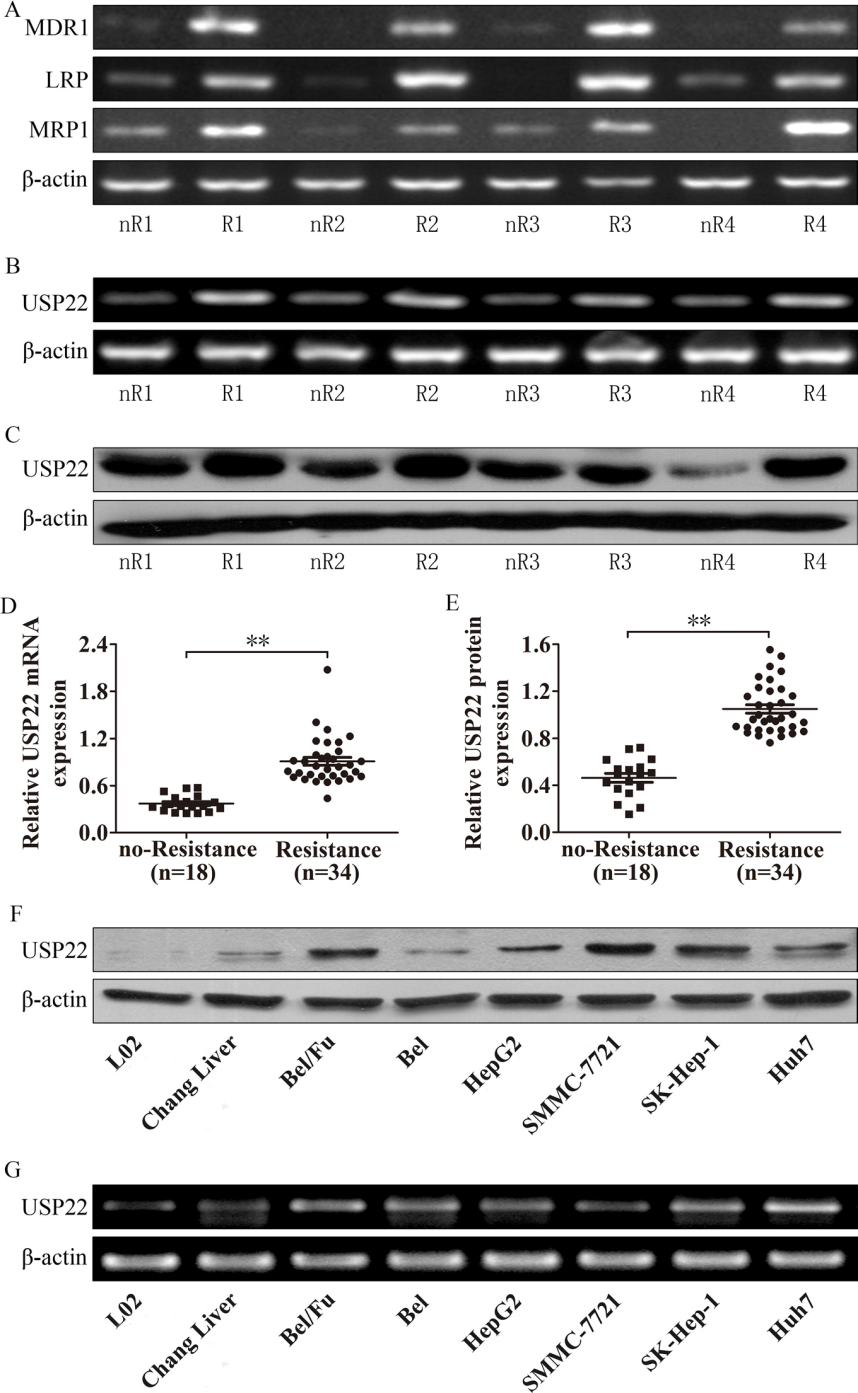
USP22 is highly expressed in HCC chemoresistance tissues and cells (**A**) MDR-related genes expression were detected by RT-PCR in HCC tissues, and these tissues were divided to chemoresistance (resistance, R) group and no-chemoresitance (no-resistance, nR) group according to the expression of MDR-related genes. (**B–E**) USP22 expression in HCC tissues. (**F–G**) USP22 expression was detected in HCC and normal liver cell lines. **P < 0.01.

To investigate whether USP22 is involved in HCC chemoresistance, the clinical data and tissues of 52 HCC patients who received TACE treatment after curative resection between 2009 and 2012 at the Second Hospital of Dalian Medical University were collected. The mRNA expression levels of MDR1, LRP and MRP1 in HCC tissues were determined using RT-PCR (Figure 1A). Based on the expression levels of MDR1, LRP and MRP1, the HCC tissues were divided into the chemoresistance (no-Resistance, nR) group and chemosensitive (Resistance, R) group. Both mRNA and protein expression of USP22 were detected in these two groups (Figure 1B and 1C). As shown in Figure 1D and 1E, the USP22 expression level in the chemoresistance group was higher than in the chemosensitive group. We also tested USP22 expression *in vivo*, and the HCC cell lines expressed higher levels of USP22 than cells of the normal liver cell line L02 (Figure 1F and 1G). Interestingly, compared with Bel-7402 (Bel) cells, USP22 expression was higher in Bel-7402/5-fluorouracil (Bel/Fu) cells.

**Figure 2:**
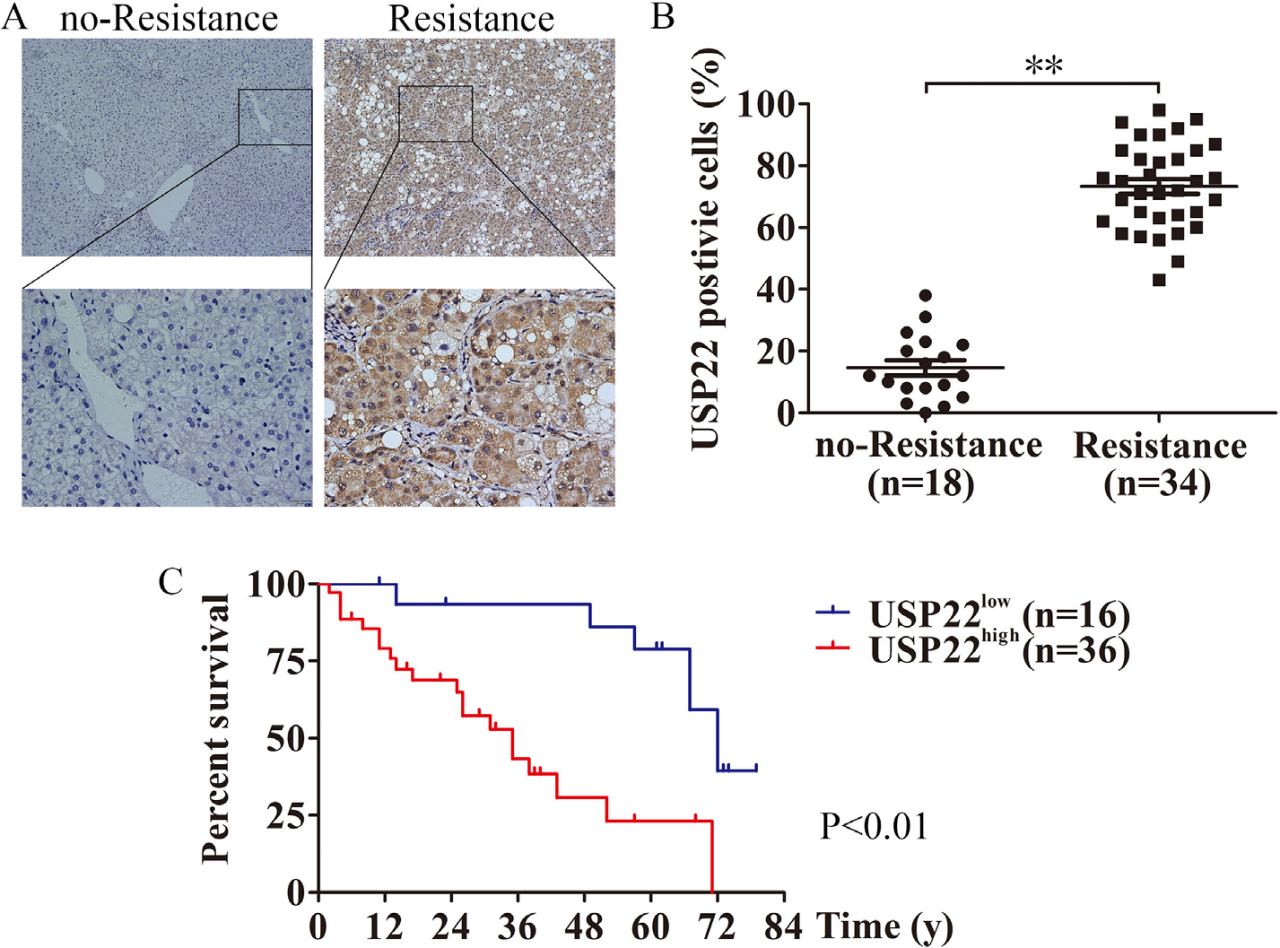
USP22 is correlated with chemoresistance in HCC (**A**) USP22 protein expression was analyzed by immunohistochemical analysis in 52 cases HCC tissues, and the representative results were shown. (**B**) Semiquantification of USP22 expression in HCC tissues without or with chemoresistance. (**C**) The association between USP22 expression in HCC and the survival time of selected patients was analyzed with Kaplan-Meier survival analysis. **P < 0.01.

We next examined USP22 protein expression in HCC samples using IHC. As expected, USP22 expression was higher in the R group than the nR group (Figure 2A and 2B). To further determine the relationship between USP22 expression and clinicopathological parameters, these 52 cases were divided into two subgroups: “low USP22 expression” and “high USP22 expression”, as defined in the immunohistochemical analysis subsection of the “materials and methods” section. Significant correlations were found between USP22 expression and the tumor size, tumor differentiation and TNM stage. There were no statistically significant differences between USP22 expression and the other clinicopathological parameters, such as patient age, gender, HBsAg and AFP (Table 1). The association between USP22 expression and survival time was analyzed using Kaplan-Meier analysis. The overall survival time of the high USP22 expression group was significantly shorter than that of the low USP22 expression group (Figure 2C). These results indicate a functional role of USP22 in the chemoresistance of HCC.

**Table 1:** USP22 staining and clinicopathologic characteristics of 52 HCC patients

Variables	USP22 staining		Total	*P*
	Low	High		
**Age (y)**				
≤ 50	**8**	**21**	**29**	
> 50	**8**	**15**	**23**	
**Sex**				
Male	**15**	**34**	**49**	
Female	**1**	**2**	**3**	
**HBsAG**				
Negative	**3**	**6**	**9**	
Positive	**13**	**30**	**43**	
**AFP (ng/ml)**				
≤ 20	**9**	**17**	**26**	
> 20	**7**	**19**	**26**	
**Tumor size (cm)**				
≤ 5	**10**	**8**	**18**	**< 0.05**
> 5	**6**	**28**	**34**	
**Tumor differentation**				
I + II	**3**	**20**	**23**	**< 0.05**
III + IV	**13**	**16**	**29**	
**TNM stage**				
I	**14**	**18**	**32**	**< 0.05**
II + III	**2**	**18**	**20**	

### USP22 knockdown inhibited growth of HCC chemoresistant cells

**Figure 3:**
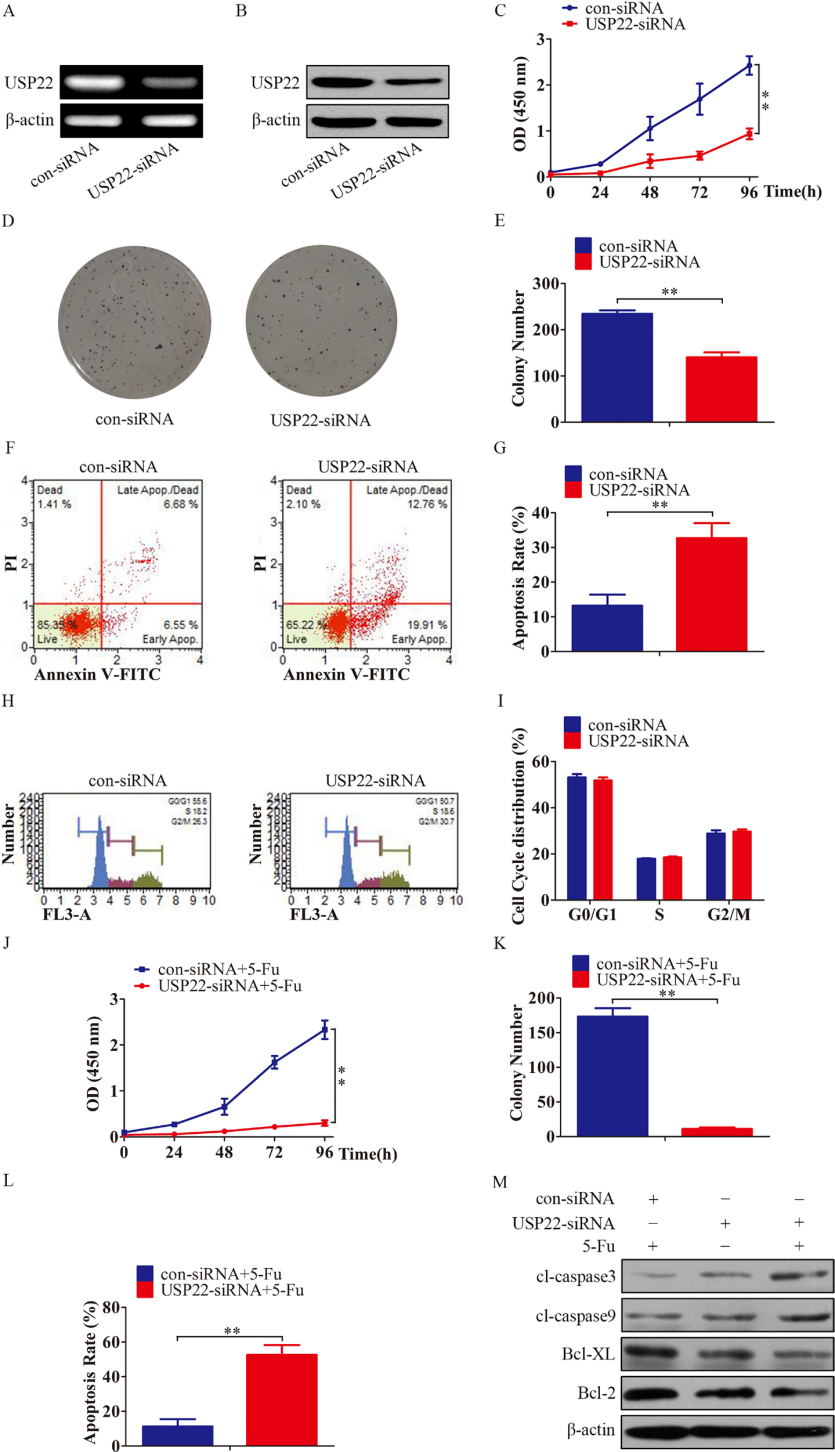
USP22 knockdown inhibits growth and promotes apoptosis of Bel/Fu cells (**A–B**) Expression of USP22 was measured after USP22-siRNA transfection by RT-PCR and western blotting. (**C**) Cell viability was measured by CCK-8 assay. (**D–E**) Cell proliferation was examined by colony formation assay. (**F–G**) The apoptotic cells were detected using Annexin V-PI dual staining. (**H–I**) After transfection, cells were stained with PI. Then, the cell cycle distribution was measured using flow cytometric analysis. (**J–L**) After combination treatment with different siRNAs transfection and 5-Fu, cell viability, colony formation and apoptosis were measured. (**K**) Cleaved caspase-3, cleaved-caspase 9, Bcl-XL and Bcl-2 levels were monitored using western blotting. **P < 0.01.

To test the functions of USP22 in HCC chemoresistance, we silenced USP22 in Bel/Fu cells, and the expression of USP22 was verified by RT-PCR and western blotting (Figure 3A and 3B). As shown in Figure 3C to 3E, silencing USP22 significantly inhibited proliferation and generated a smaller number of colonies in Bel/Fu cells. To further understand the role of USP22 in the control of HCC chemoresistant cell growth, we performed flow cytometry. Silencing USP22 promoted Bel/Fu cell apoptosis (Figure 3F and 3G), but had no effect on cell cycle progression (Figure 3H and 3I). We also found that USP22 knockdown enhanced the anti-growth and pro-apoptotic effect of 5-Fu in Bel/Fu cells (Figure 3J–3L). USP22 knockdown also decreased the expression of Bcl-XL and Bcl-2 and increased the expression of cleaved-caspase 3 and cleaved-caspase 9. In addition, USP22 knockdown combined with 5-Fu treatment further enhanced the down-regulation of Bcl-XL, Bcl-2 and up-regulated cl-caspase 3 and cleaved-caspase 9 (Figure 3M).

**Figure 4:**
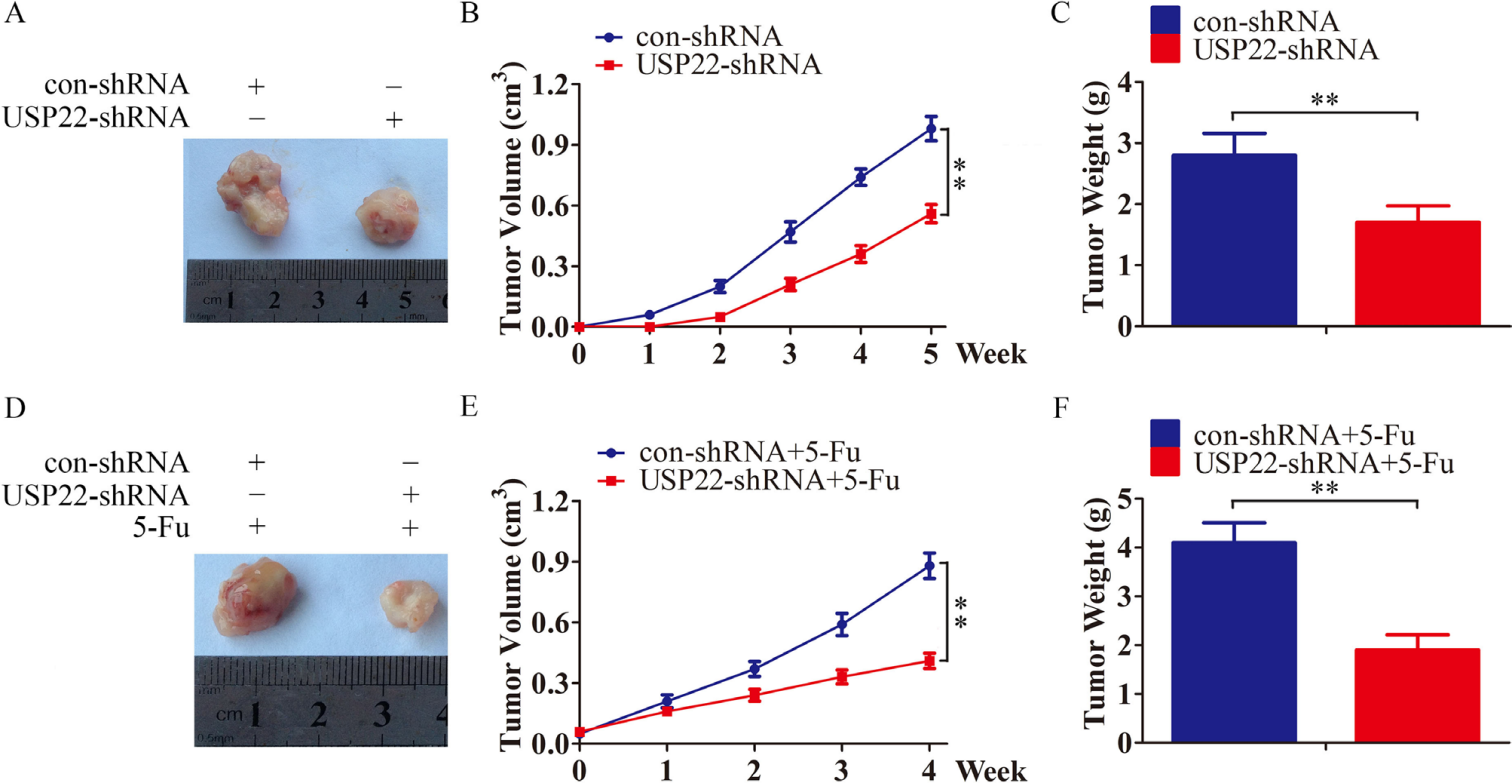
USP22 knockdown inhibits tumorigenesis and chemoresistance *in vivo*. (**A, D**) Representative images of subcutaneous tumors formed by Bel/Fu-con-shRNA and Bel/Fu-USP22-shRNA cells (A), or the corresponding cells treated with 5-Fu (D). (B, E) Growth curve of tumors formed by Bel/Fu-con-shRNA and Bel/Fu-USP22-shRNA cells (B), or the corresponding cells treated with 5-Fu (**E**). (**C, F**) Weight of tumors formed by Bel/Fu-con-shRNA and Bel/Fu-USP22-shRNA cells (B), or the corresponding cells treated with 5-Fu (E) at harvest time. **P < 0.01.

Bel/Fu-control cells and Bel/Fu-USP22-shRNA cells were subcutaneously injected into nude mice. The tumor size was measured every week for up to 5 weeks. Consistent with the *in vitro* observations, silencing USP22 led to a dramatic decrease in tumor volume and weight compared with the control cells (Figure 4A–4C), and also enhanced chemosensitivity to 5-Fu *in vivo* (Figure 4D–4F). Taken together, these results suggest that USP22 is an important regulator of growth and chemoresistance in HCC chemoresistant cells.

### USP22 knockdown suppressed migration and invasion of HCC chemoresistant cells

**Figure 5:**
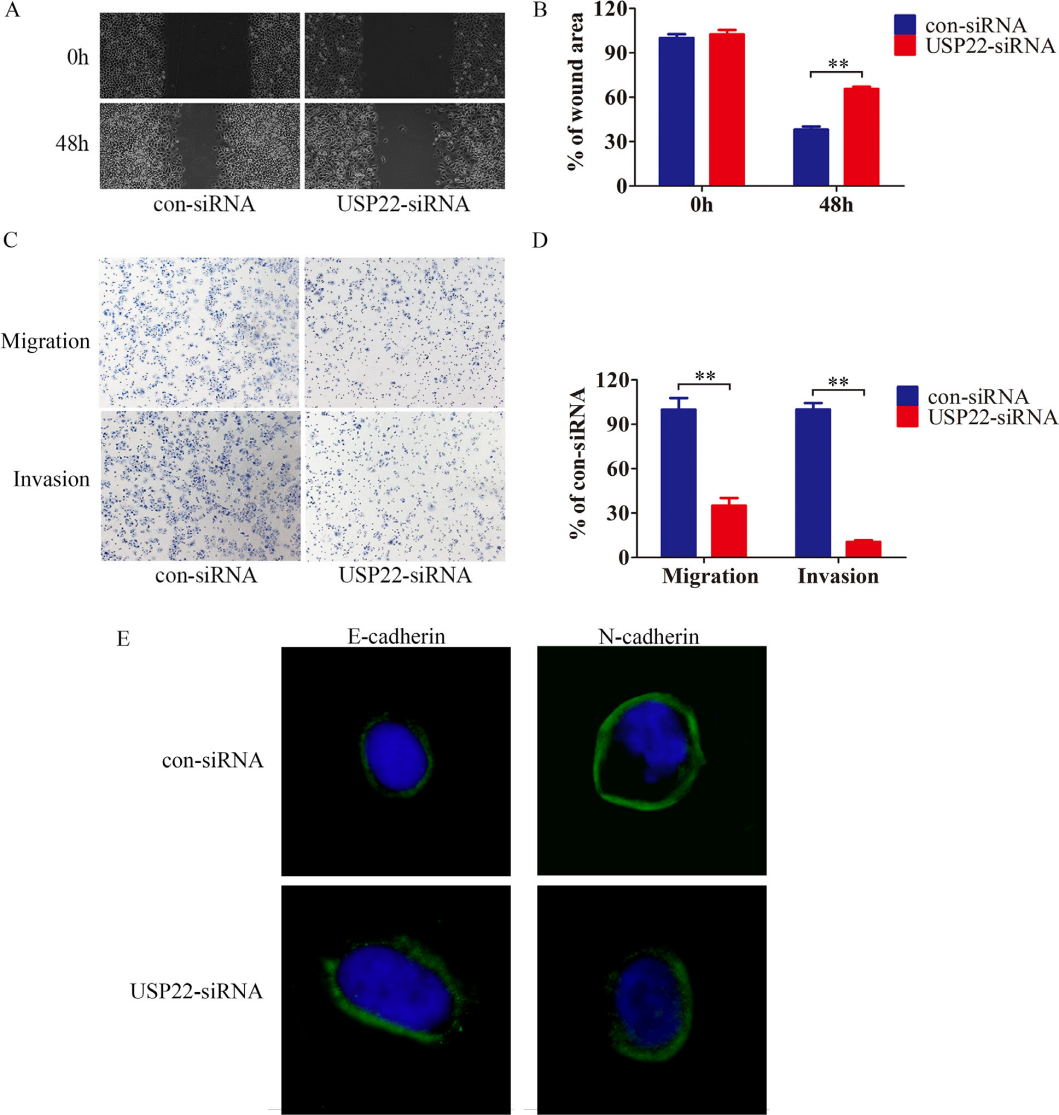
USP22 knockdown suppressed migration, invasion and EMT of HCC chemoresistant cells (**A–B**) Bel/Fu-control-siRNA and Bel/Fu-USP22-siRNA cells were subjected to wound healing assay, the uncovered area was quantified as a percentage of the original wound area. (**C–D**) Bel/Fu-control-siRNA and Bel/Fu-USP22-siRNA cells were subjected to migration and invasion assay, quantification of migrated and invaded cells through the membrane are shown as proportions of Bel/Fu-con-siRNA cells. (**E**) Expression of epithelial and mesenchymal markers were analyzed by immunofluorescence. **P < 0.01.

Next, we assessed the effect of USP22 on migration and invasion of HCC chemoresistant cells. Silencing USP22 decreased the migration and invasion of Bel/Fu cells, and combination with 5-Fu further decreased the rate of migration and number of invading cells (Figure 5A–5D).

EMT has been confirmed to be a key contributor to tumor migratory and invasive capacities. We investigated whether USP22 exerts the anti-migration and anti-invasion activities that accompany EMT regulation. The assessment of EMT in cell lines has been reported based on the analysis of changes in the expression of molecular markers, including E-cadherin and N-cadherin [17, 18]. As shown in Figure 5E, the expression of the epithelial marker E-cadherin was higher in USP22 silenced cells than in the control cells. In contrast, the mesenchymal marker, N-cadherin, was decreased in USP22 silenced cells. Therefore, these findings suggest that USP22 promotes migratory and invasive behaviors in HCC chemoresistant cells.

### USP22 knockdown decreased MDR-related genes expression through up-regulation of Smad4 and suppression of Akt

**Figure 6:**
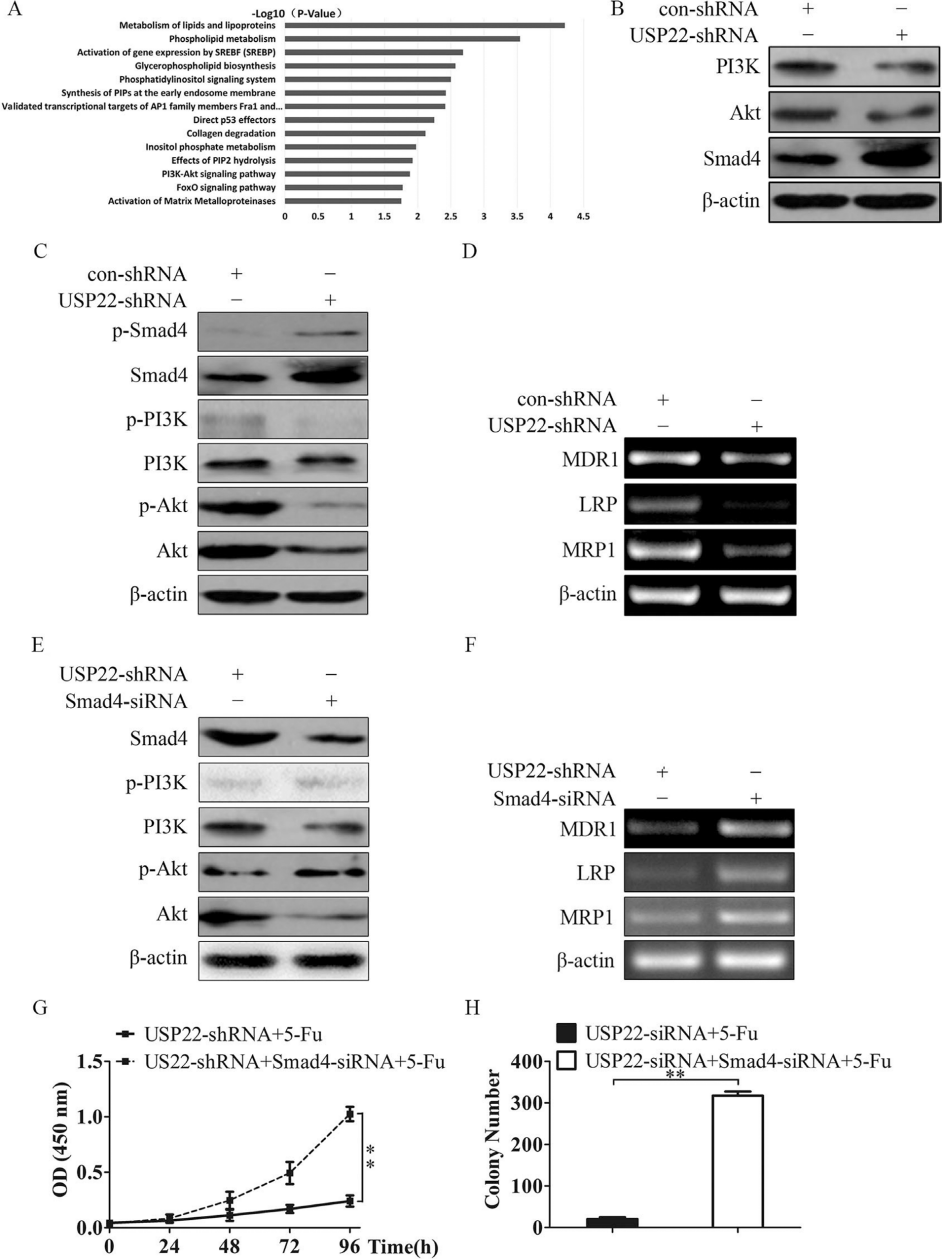
USP22 knockdown blocks phosphorylation of Akt via down-regualting PI3K and activating Smad4 (**A**) Gene set enrichment analysis was carried out using ConceptGen. (**B**) Protein levels of PI3K, Akt and Smad4 were measured in Bel/Fu-control-shRNA and Bel/Fu-USP22-shRNA cells by western blotting. (**C, E**) Phosphorylated protein and protein levels of PI3K, Akt and Smad4 were measured by western blotting. (**D, F**) MDR-related genes were measured by RT-PCR. (**G**) Cell viability was measured by CCK-8 assay. (**H**) Cell proliferation was examined by colony formation assay. **P < 0.01.

To better understand the mechanisms by which USP22 is involved in the development of chemoresistance in HCC, we performed gene expression profiling on Bel/Fu-USP22-shRNA cells and control cells. Microarray analyses identified a list of genes that were significantly differentially expressed after USP22 knockdown, including down-regulation of PI3K, Akt and up-regulation of Smad4 (data not shown). Gene set enrichment analysis indicated that proliferation, motility, cell movement and invasion related gene signatures were significantly changed in USP22 silenced cells, including the PI3K/Akt pathway (Figure 6A), supporting the idea that USP22 regulates proliferation, migration and invasion of Bel/Fu cells. We verified PI3K, Akt and Smad4 expression by western blotting, and the results agreed with those of the microarray analyses (Figure 6B). These data led us to hypothesize that USP22 regulates these functions possibly via regulating Smad4 and the PI3K/Akt pathway.

We compared the expression of USP22 and Smad4 with clinicopathological parameters in these 52 HCC cases. Patients with high expression of USP22 and low expression of Smad4 significantly has lower AFP, smaller tumor size and higher TNM stage (Table 2).

**Table 2:** Combination of USP22 and Smad4 expression and their correlation with clinicopathologic characteristics of 52 HCC patients

UPS22 expression	Low	Low	High	High	*P*
Smad4 expression	High	Low	High	Low	
Total	5	11	9	27	
**Age (y)**					
≤ 50	**3**	**5**	**4**	**17**	
> 50	**2**	**6**	**5**	**10**	
**Sex**					
Male	**5**	**10**	**7**	**27**	
Female	**0**	**1**	**1**	**0**	
**HBsAG**					
Negative	**2**	**1**	**3**	**3**	
Positive	**3**	**10**	**6**	**24**	
**AFP (ng/ml)**					
≤ 20	**1**	**10**	**8**	**9**	**< 0.01**
> 20	**4**	**1**	**1**	**28**	
**Tumor size (cm)**					
≤ 5	**4**	**6**	**4**	**4**	**< 0.01**
> 5	**1**	**5**	**5**	**23**	
**Tumor differentation**					
I + II	**1**	**2**	**5**	**15**	**< 0.05**
III + IV	**4**	**9**	**8**	**8**	
**TNM stage**					
I	**4**	**10**	**7**	**11**	**< 0.05**
II + III	**1**	**1**	**2**	**16**	

We next examined whether Smad4 and PI3K/Akt are crucial for USP22 regulated chemoresistance in HCC cells. Compared with Bel/Fu-con-shRNA cells, silencing UPS22 inhibited the phosphorylation of PI3K and Akt, enhanced epxression of Smad4 and promoted phosphorylation of Smad4 (Figure 6C). mRNA expression of MDR-related genes were also decreased after USP22 konckdown (Figure 6D). By contrast, silencing Smad4 rescued the impaired phosphorylation of Akt and the MDR-related genes levels in Bel/Fu-USP22-shRNA cells, but has no effect on p-PI3K (Figure 6E and 6F). And silencing Smad4 made Bel/Fu-USP22-shRNA cells re-chemoresistance to 5-Fu (Figure 6G and 6H).

These results suggest that USP22 knockdown-induced chemosensitivity of HCC cells by down-regualting PI3K and Akt, and Smad4-mediated Akt suppression as well.

## DISCUSSION

HCC easily acquires chemoresistance. Thus, conventional chemotherapy treatments achieve poor efficacy in patients with advanced HCC and show little benefit to overall survival. Deubiquitination is the process of removing ubiquitin from a substrate. As a crucial type of post-translational modification, deubiquitination is involved in diverse biological behaviors, including regulation of protein activity and cellular homeostasis. A disturbed balance between ubiquitination and deubiquitination also results in a variety of pathologic processes [19, 20]. USP22 is a novel human deubiquitinating enzyme. USP22 may deubiquitinate H2A and H2B, subunits of the hSAGA complex that activate transcription factors and promote carcinogenesis [21]. Our previous work has demonstrated that increased expression of USP22 in HCC might be important for tumor progression and can serve as an independent biomarker for poor survival [22, 23]. However, it remains unclear whether USPP22 is involved in HCC chemoresistance; the underlying mechanism is also unknown.

**Figure 7:**
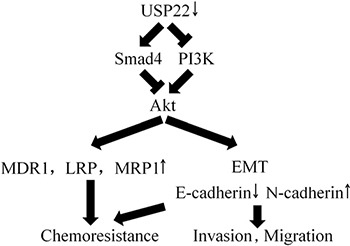
Schematic model of the role of USP22 in HCC chemoresistance

To the best of our knowledge, this is the first study to identify the effect of USP22 on chemoresistance in HCC. We found that USP22 expression was higher in HCC chemoresistant tissues than in chemosensitive ones, and a high USP22 expression was correlated with a poor prognosis. Silencing USP22 in Bel/Fu cells inhibited proliferation, migration, invasion and chemoresistance both *in vitro* and *in vivo*. We also found that USP22 exerted its function through PI3K/Akt pathway. Silencing Smad4 reversed the inhibition of Akt and MDR-related genes caused by USP22 knockdown in Bel/Fu cells (Figure 7).

5-Fu can interfere with nucleoside metabolism and result in DNA synthesis disorders and RNA dysfunction, leading to cytotoxicity and cell death and has already been used in a variety of tumors [24]. Many factors have been identified in the development of resistance to chemotherapeutic agents, such as elevated expression of drug efflux transporters; changes in drug kinetics; amplification of drug targets or the transition from epithelial to mesenchymal-like cells, which comprises genetic variation; and the tumor microenvironment [25]. In our study, when we silenced USP22 in Bel/Fu cells, the expression of MDR1, LRP and MRP1 decreased and cells transitioned from mesenchymal to epithelial-like cells, leading to enhanced sensitivity to 5-Fu. These results may be the reason why high USP22 expression indicates insensitivity to chemotherapeutic agents, tendency to metastasis and a poor prognosis in HCC.

The PI3K pathway comprises a family of intracellular signal transducer enzymes with three key regulatory nodes: PI3K, AKT, and mammalian target of rapamycin (mTOR). PI3Ks are heterodimeric lipid kinases that are composed of a regulatory subunit and a catalytic subunit that are encoded by different genes. One of the main functions of PI3K is to synthesize the secondary messenger phosphatidylinositol triphosphatase (PIP3) from phosphatidylinositol biphosphate (PIP2). The generation of PIP3 activates PI3K, following Akt recruitment at the inner leaflet of the plasma membrane. At the membrane, another PH-domain containing serine/threonine kinase, named 3-phosphoinositide-dependent protein kinase-1 (PDK1), phosphorylates Akt [26]. Akt activation by phosphorylation regulates critical cellular activities, such as protein synthesis, growth, differentiation, metabolism, and survival, as well as tumorigenesis [26, 27]. Our present study identified a list of genes differentially expressed after USP22 knockdown, including Smad4 and genes of the PI3K/Akt pathway. Silencing Smad4 reversed the inhibition of Akt and MDR-related proteins according to USP22 knockdown. Therefore, we suggest that USP22 regulates the chemoresistance of HCC by Smad4/Akt-dependent MDR-related genes modulation.

In summary, USP22 knockdown results in the up-regulation of epithelial marker and the down-regulation of mesenchymal marker, thereby preventing Bel/Fu cells from becoming metastatic. Moreover, USP22 knockdown also decreases MDR-related genes expression by inhibiting Akt phosphorylation, and USP22 knockdown mediated Smad4 up-regulation is crucial for Akt suppression. Both the regulation of EMT and the expression of MDR-related genes are responsible for the enhanced sensitivity to 5-Fu *in vitro* and *in vivo*. Therefore, we suggest USP22 to be a new therapeutic target for improving the efficacy of chemotherapy in HCC patients.

## MATERIALS AND METHODS

### Patients and specimens

Tumor tissues that were used for RT-PCR and western blotting were collected from 52 HCC patients who underwent TACE after curative resection with informed consent between 2009 and 2011 at the Second Hospital of Dalian Medical University. Tumor staging was based on the 6th edition of the tumor-node-metastasis (TNM) classification of the International Union Against Cancer. Follow-up data were summarized at the end of March 2016, with a median observation time of 34.4 months. The study protocols were approved by the Hospital Ethics Committee of the Second Hospital of Dalian Medical University. Written informed consent based on the Declaration of Helsinki was obtained from the patients.

### Immunohistochemical analysis

Human tumor tissues were fixed in 4% paraformaldehyde overnight and subsequently embedded in paraffin wax. Sections were cut at a thickness of 4 μm and were stained with hematoxylin and eosin for histological analysis. Immunohistochemical analysis was performed for different markers in arrays as previously described [28]. The proportion of stained cells (lower, < 30% staining; higher, ≥ 30% staining) was semiquantitatively determined following published protocols [29].

### Cell lines and cell culture

The normal human liver cell line L02 and the HCC cell lines HepG2, SK-Hep-1, Li-7, Huh7, SMMC-7721, and Bel-7402 were purchased from the American Type Culture Collection (ATCC; Danvers, MA, USA). Cells from the lines L02, HepG2, SK-Hep1, Huh7, and SMMC-7721 were cultured in DMEM medium (Invitrogen, Carlsbad, CA, USA). Li-7 and Bel-7402 cells were cultured in 1640 medium (Invitrogen). To obtain 5-Fu-resistant Bel-7402 (Bel/Fu) cells, Bel-7402 cells were cultured in RPMI-1640 medium supplemented with 1.0 × 10-7 mol/l 5-Fu (Sigma-Aldrich, St. Louis, MO, USA) for 6 months. Once the drug resistance assessment was successful, the cells were cultured in 1640 medium. All media were supplemented with 10% FBS (Invitrogen). Cells were incubated at 37°C in a humidified atmosphere containing 5% CO2.

### Establishment of USP22 knockdown and Smad4 stable expression cell lines

Lentiviral vector-mediated RNA interference was used to infect Bel/Fu cells as a negative control, and a USP22-special shRNA lentivirus was prepared and used to infect Bel/Fu cells as previously described [30]. Bel/Fu cells expressing Smad4 were established as previously described [31]. The expression of USP22 and Smad4 was confirmed by western blotting analysis.

### Cell viability assay

Cells were seeded into 96-well plates in triplicate at densities of 3000 per well. Cell viability was monitored at desired time points using the Cell Counting Kit-8 (CCK8, KeyGen Biotech, Nanjing, China) according to the manufacturer's instructions.

### Colony formation assay

A total of 500 cells/well were seeded into 6-well plates in triplicate. After 2 weeks of growth, the cells were fixed and stained with crystal violet. Visible colonies were counted according to the cell numbers in each colony.

### Flow cytometric analysis

Cells were seeded into 6-well plates (2×10^5^ cells/well). For cell cycle analysis, the cells were harvested and resuspended in 2 ml of ice-cold 70% ethanol at 4°C and left overnight. The fixed cells were centrifuged and washed with PBS. After incubation with 100 μl of RNase A (10 μg/ml) for 30 min at 37°C, cells were resuspended in 400 μl of PI (50 μg/ml) and placed in the dark at room temperature for 30 min. For apoptosis analysis, the cells were digested and resuspended in binding buffer to prepare single cell suspensions. Then, the cells were stained using the annexin V-FITC reaction reagent (5 μl of annexin V-FITC, 5 μl of propidium iodide) and incubated in the dark at room temperature for 30 min. Then, the stained cells were analyzed.

### *In vivo* tumor growth

Nude mice were purchased from the SPF Laboratory Animal Center at Dalian Medical University. All animals were used in accordance with institutional guidelines, and the current experiments were approved by the Animal Care and Use Committee. Bel/Fu-Con-shRNA cells (1 × 10^7^/ 100 μl) were resuspended in PBS and inoculated subcutaneously into 6 nude mice that were 4 weeks old. In a similar procedure, Bel/Fu-USP22-shRNA cells were inoculated into 18 nude mice that were 4 weeks old. Two weeks later, when the tumor diameters had reached 4 mm to 5 mm, 12 of the Bel/Fu-USP22-shRNA inoculated mice were randomly divided into 2 groups (*n* = 6/group) that were administered either 30 mg/kg 5-Fu in the 5-Fu group or normal saline in the NS group. These agents were intraperitoneally injected three times per week for six weeks. The tumors were measured weekly and the tumor volume was calculated according to the formula length × width^2^/2. The mice were killed at six weeks after inoculation, and the weight of the tumors in each mouse was measured.

### Wound healing assay

Cells were seeded into 6-cm culture plates. The cell monolayers were wounded using 200-μl micropipette tips and photographed using microscopy at desired time points. The migration distance of each cell was measured by Image-pro Plus.

### Cell invasion and migration assay

The invasion of cells was measured in matrigel-coated (BD, Franklin Lakes, NJ, USA) transwell inserts (6.5 mm, Costar, Manassas, VA, USA) containing polycarbonate filters with 8-μm pores. The inserts were coated with 50 μl of 1 mg/ml Matrigel matrix according to the manufacturer's recommendations. A quantity of 2 × 10^5^ cells in 200 μl of serum-free medium were plated in the upper chamber, whereas 300 μl of medium with 10% fetal bovine serum were added to the lower well. After 24 h incubation, the cells that invaded the lower surface of the membrane were fixed and stained. Five random fields were counted at ×10 magnification for each membrane. Migration assays were similar to invasion assay except that the transwell insert was not coated with matrigel.

### Immunofluorescence staining

Cells were fixed with 4% paraformaldehyde and permeabilized with 0.1% Triton X-100. Blocked with 5% bovine serum albumin (BSA), followed by the primary and second antibodys sequentially. The nuclei were stained with DAPI. The images were captured using a fluorescence microscopy.

### RT-PCR

Total RNA was isolated using the RNAiso™ Plus kit (Takara, Japan). Total RNA (500 ng) was reverse-transcribed into first-strand cDNA using a Takara RNA PCR kit AMV Ver. 3.0 (Takara Biotechnology, Dalian, China) according to the manufacturer's instructions. The following primers were used for amplification: MDR1, forward primer 5′-CCCATCATTGCAATAGC AGG-3′ and reverse primer 5′-GTTCAAACTTCTGCTCC TGA-3′; LRP, forward primer 5′-TTTCAGTGCCAGACTT TGTAG-3′ and reverse primer 5′-GATGCGGGCTGAG TTCTTATG-3′; MRP1, forward primer 5′-TGAAGGACTT CGTGTCAGCC-3′ and reverse primer 5′-GTCCATGAT GGTGTTGAGCC-3′; USP22, forward primer 5′-GGCG GAAGATCACCACGTAT-3′ and reverse primer 5′-TTGTT GAGACTGTCCGTGGG-3′; and β-actin, forward primer 5′- GCATGGAGTCCTGTGGCAT-3′ and reverse primer 5′- CTAGAAGCATTTGCGGTGG -3′. The PCR reactions were subjected to the amplification protocol, as previously described [36].

### Western blotting

The cells were lysed in RIPA buffer on ice supplemented with 1 mM PMFS and 1 mM phosphatase inhibitor cocktail to obtain total cellular protein. Protein concentrations were determined using a BCA protein assay kit. The protein samples (30 μg) were mixed with loading buffer, separated by 10% SDS-PAGE and transferred onto PVDF membranes. The membranes were then blocked and incubated with the primary and secondary antibodies. Then, the bands were visualized by chemiluminescence.

### Gene expression profiling

Total RNA quality and quantity were determined using Agilent 2100 Bioanalyzer and NanoDrop ND-1000. Affymetrix HU U133 plus 2.0 arrays were used according to the manufacturer's protocol. The data were initially normalized by robust multiarray average (RMA) normalization algorithms in expression console software (Affymetrix). Significantly altered genes between USP22 knockdown and its control cells were considered by scatter plots, and the genes were up- and downregulated ≥ 5-fold. Clustering analysis was performed using the gene list by Gene Cluster v3.0 software. Gene set enrichment analysis was conducted using ConceptGen (http://conceptgen.ncibi.org/core/conceptGen/index.jsp). Gene sets were obtained either from ConceptGen or from published gene signatures.

### Statistical analysis

The correlations between USP22 expression and age, sex, tumor size, TNM stage, and other clinicopathologic parameters (Table 1) were evaluated using a chi-square (χ2) test. The correlations of clinicopathologic parameters among the four groups were evaluated using fisher's exact test. The survival probability was estimated using the Kaplan-Meier method, and the significant difference between high USP22 and low USP22 groups was determined using the log-rank test. Comparisons among multiple groups were made with a one-way analysis of variance (ANOVA) followed by Dunnet *t-test*. All experiments were repeated three times. Data were expressed as the mean ± SD and were analyzed using the Student's *t-test*. The statistical significance of the differences between mean values was determined by *P* < 0.05. SPSS 16.0 software was used for all statistical analysis.
